# Geographic distribution and risk of upper urothelial carcinomas in Croatia, 2001–2011

**DOI:** 10.1186/s12885-019-6160-9

**Published:** 2019-10-15

**Authors:** Danira Medunjanin, Zdenko Sonicki, John E. Vena, Ante Cvitkovic, Sara Wagner Robb

**Affiliations:** 1 Department of Public Health Sciences, Medical University of South Carolina, Charleston, SC USA; 2 University of Zagreb, School of Medicine, Andrija Stampar School of Public Health, Zagreb, Croatia; 3 Institute for Public Health, Brodsko Posavska County, Slavonski Brod, Croatia; 4 Josip Juraj Strossmayer University of Osijek, Faculty of Medicine, Osijek, Croatia; 5 Josip Juraj Strossmayer University of Osijek, Faculty of Dental Medicine and Health, Osijek, Croatia; 6 Department of Public Health Sciences, Clemson University, Clemson, SC USA

**Keywords:** Upper urothelial carcinoma, Balkan endemic nephropathy, Standardized incidence ratio, Geographic information system

## Abstract

**Background:**

Strong associations exist between Balkan endemic nephropathy (BEN) and upper urothelial carcinomas (UUCs). However, the common etiology between the two remains unclear and there are no studies to date that visualize UUC risks in Croatia. In Croatia, 14 villages in the southwestern part of Brod-Posavina County are considered endemic for BEN. The aim of this ecological study is to map cancer risks and describe the case distribution of UUCs in Croatia at the county level during 2001–2011.

**Methods:**

A total of 608 incident cases from the Croatian National Cancer Registry were identified. Indirect standardization was employed to compute standardized incidence ratios (SIRs).

**Results:**

Counties with SIRs greater than 1 were concentrated around the agricultural region of Slavonia and the coastal region of Dalmatia. However, only Brod-Posavina County and Vukovar-Srijem County had a statistically significant risk of UUC development, where there were 390 and 210% more UUC cases observed than expected, respectively. Only unique to Brod-Posavina County, females were at higher risk (SIR 4.96; 95% CI 3.59–6.34) of developing UUCs than males (SIR 3.03; 95% CI 2.04–4.01) when compared to their Croatian counterparts. Although Brod-Posavina County only made up 3.7% of the total Croatian population (as of 2011), it had the highest frequency of incident UUC cases after the capital City of Zagreb. No elevated cancer risks were noted in the City of Zagreb, even after stratifying by sex.

**Conclusion:**

Our findings suggest that Brod-Posavina County had the highest cancer risk for UUCs, especially among females, when compared to Croatia as a whole during 2001–2011. Given that a majority of BEN patients develop associated UUCs, concurrent screening programs for UUCs and BEN should be considered not only in endemic areas of BEN but also the surrounding rural areas and amongst at-risk groups such as those undergoing hemodialysis, who frequently develop UUCs, to help clarify BEN-UUC associations by identifying common risk factors while standardizing disease estimates across endemic regions for BEN.

## Background

Urothelial carcinomas (UCs) are malignancies of the transitional epithelium that lines the inner surface of the urinary organs including the bladder, urethra, ureter, and renal pelvis [1]. There are two types of UCs; they can either be located in the lower (bladder, urethra) or upper (ureter, renal pelvis) urinary tract [2]. Tumors of the bladder are the most common type; they make up 90–95% of all UCs [1, 2]. In contrast, UCs of the upper urinary tract only account for 5–10% of all UC cases [1–3]. Although rare, 60% of upper urothelial carcinomas (UUCs) are invasive at diagnosis compared to tumors of the bladder, of which, only 15–25% are invasive at diagnosis [2]. Moreover, UUCs are more common among people in their senior years (70–90 years of age) and are 2 to 3 times more prevalent among men than women [2, 4, 5].

The annual incidence of UUCs in Western countries is about 1–2 cases per 100,000 [2, 4, 5]. Higher frequencies of UUCs have been shown in endemic regions for Balkan endemic nephropathy (BEN), a chronic tubulointerstitial disease that is endemic along the tributaries of the Danube River in countries such as Bulgaria, Bosnia and Herzegovina, Serbia, Romania, and Croatia, as early as 40 to 50 years ago [6–13]. In Croatia, 14 villages in the southwestern part of Brod-Posavina County, located in the flood plains of the Sava River, are considered endemic for BEN [9, 14]. Recent findings from several studies suggest a decline in BEN incidence [15–19]; however, conflicting results still exist [20–23]. As for UUC incidence, it appears to be declining according to Markovic et al., who reported a decrease in UUCs over a 30-year period when endemic areas for BEN were compared to non-endemic areas in Serbia (57.1-fold in 1969–1988 vs. 11.2-fold in 1989–1998) [15]. Nonetheless, the risk of UUCs remained high in the BEN endemic areas for 1989–1998 [15].The common etiology between the association of UUCs and BEN remains unclear and there are no studies to date that visualize UUC risks in Croatia. This ecological study aims to map cancer risks and describe the case distribution of UUCs in Croatia at the county level during 2001–2011 using a geographic information system (GIS). Additional comparisons between Brod-Posavina County and the City of Zagreb were made to determine the characteristics of UUCs in endemic and non-endemic counties for BEN.

## Methods

County-level data on the number of newly reported UUC cases obtained from hospital discharge records and notifications from outpatient clinics with histological and cytological findings between 2001 and 2011 through the Croatian National Cancer Registry were analyzed. A total of 608 incident UUC cases from 20 counties (i.e., Bjelovar-Bilogora, Brod-Posavina, Dubrovnik-Neretva, Istria, Karlovac, Koprivnica-Krizevci, Krapina-Zagorje, Lika-Senj, Medimurje, Osijek-Baranja, Pozega-Slavonia, Primorje-Gorski Kotar, Sibenik-Knin, Sisak-Moslavina, Split-Dalmatia, Varazdin, Virovitica-Podravina, Vukovar-Srijem, Zadar, and Zagreb County) and the capital City of Zagreb were obtained. Of these Croatian counties, the following five, Brod-Posavina, Osijek-Baranja, Pozega-Slavonia, Virovitica-Podravina and Vukovar-Srijem County correspond to the historical region of Slavonia located east of the country. A UUC case was defined and coded according to the International Classification of Diseases 10th Revision (ICD-10) as any malignant neoplasm of either the renal pelvis (C65), ureter (C66), or any other and unspecified urinary organ (C68). To note, we were unable to distinguish between histological types. However, over 95% of urinary tract tumors are of the urothelium [24]. The data were stratified according to age, sex, and diagnosis. Age at diagnosis was categorized into 5-year age groups that ranged from 0 to 85 and above while diagnosis was organized into three categories (i.e. the renal pelvis, ureter, and other urinary organs) based on ICD-10 codes.

For descriptive purposes, age at diagnosis was categorized into 4 categories: < 60, 60–69, 70–79, and ≥ 80. Due to limitations in sample size amongst the age strata for each county (*n* < 20), study years were combined (2001–2011). The indirect standardization method was used to estimate the standardized incidence ratio (SIR) with a 95% confidence interval (CI) for each county by dividing the total number of observed cases by the total number of expected cases. Age-specific UUC reference rates, as per the 2011 Croatian Census from the Croatian Bureau of Statistics, were used in estimating the total number of expected cases. SIRs were stratified by county and sex. A *p*-value of less than 0.05 was considered statistically significant. All results were computed using SAS 9.4 (Cary, NC: SAS Institute Inc.). A map was generated to display SIRs at the county-level using QGIS version 3.2.0.

## Results

A total of 608 UUC cases were identified in Croatia during 2001–2011, of which, 55.4% were males (Table 1). Conversely, in Brod-Posavina County incident UUC cases were predominantly female (58.1%). More than half (61.6%) of all UUC cases in Brod-Posavina County and 43.3% in Croatia were in their 70s at the time of diagnosis. In the City of Zagreb, UUC cases were significantly younger (*p* = 0.0001) than those in Brod-Posavina County.

**Table 1:** Characteristics of incident upper urothlial carcinoma cases in Croatia, Brod-Posavina County, and the City of Zagreb, 2001-2011

	Croatia	Brod-Posavina County	City of Zagreb	*p*-value^a^
N	608	86	107	
Sex *% Male*	55.4	41.9	55.1	0.0823
Age at Diagnosis *n (%)*				
< 60	105 (17.3)	6 (7.0)	22 (20.5)	0.0001
60–69	170 (27.9)	15 (17.4)	37 (34.6)	
70–79	263 (43.3)	53 (61.6)	34 (31.8)	
≥ 80	70 (11.5)	12 (14.0)	14 (13.1)	
Diagnosis *n (%)*				
Renal Pelvis	325 (53.4)	43 (50.0)	57 (53.3)	0.7970
Ureter	206 (33.9)	29 (33.7)	36 (33.6)	
Other Urinary Organs	77 (12.7)	14 (16.3)	14 (13.1)	

^a^ Fisher Exact test two-sided p-value comparing Brod-Posavina County to City of Zagreb

Fifty-three percent of all UUC cases in Croatia were tumors of the renal pelvis followed by the ureter (33.9%) and other urinary organs (12.7%). No differences in diagnoses were noted between Brod-Posavina County and the City of Zagreb.

**Fig. 1:**
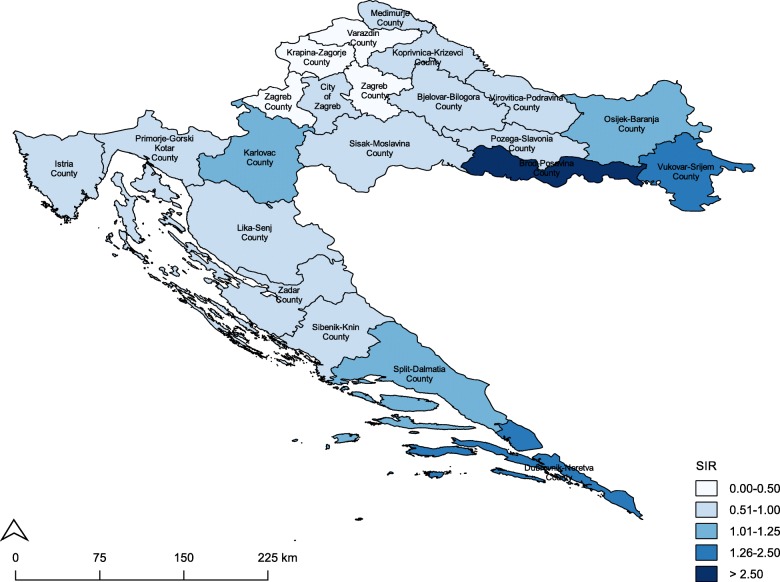
Map of standardized incidence ratios for upper urothelial carcinomas in Croatia by county and the City of Zagreb, 2001-2011

The City of Zagreb had the highest frequency of incident UUC cases in 2001–2011 followed by Brod-Posavina County and Split-Dalmatia County (Table 2). Counties with SIRs greater than 1 were concentrated around the eastern agricultural region of Slavonia and the southern coastal region of Dalmatia (Fig. 1). However, only two Slavonian counties, Brod-Posavina County and Vukovar-Srijem County, had a statistically significant risk of UUC development, where there were 390 and 210% more UUC cases observed than expected, respectively. Moreover, males and females in Brod-Posavina County had a 3-fold and 5-fold increased UUC risk compared to their Croatian counterparts (Table 3). No elevated cancer risks were observed in the City of Zagreb, even after stratifying by sex.

**Table 2:** Standardized incidence ratios for upper urothelial carcinomas by county and the City of Zagreb, 2001-2011

	Observed	Expected	SIR (95% CI)
Brod-Posavina County^c^	86	22.1	3.90 (3.08, 4.72)^b^
Vukovar-Srijem County^c^	52	24.8	2.10 (1.53, 2.67)^a^
Dubrovnik-Neretva County	22	17.4	1.27 (0.74, 1.80)
Split-Dalmatia County	68	61.3	1.11 (0.85, 1.37)
Karlovac County	23	21.0	1.10 (0.65, 1.54)
Osijek-Baranja County^c^	45	42.0	1.07 (0.76, 1.38)
City of Zagreb	107	108.8	0.98 (0.80, 1.17)
Virovitica-Podravina County^c^	11	11.8	0.93 (0.38, 1.48)
Primorje-Gorski Kotar County	38	45.6	0.83 (0.57, 1.10)
Istria County	25	30.4	0.82 (0.50, 1.15)
Sisak-Moslavina County	22	26.8	0.82 (0.48, 1.17)
Pozega-Slavonia County^c^	9	11.1	0.81 (0.28, 1.35)
Zadar County	20	25.0	0.80 (0.45, 1.15)
Medimurje County	10	14.4	0.69 (0.26, 1.12)
Koprivnica-Krizevci County	11	16.4	0.67 (0.27, 1.07)
Lika-Senj County	6	9.30	0.64 (0.13, 1.16)
Bjelovar-Bilogora County	11	17.6	0.63 (0.26, 0.99)^a^
Sibenik-Knin County	10	18.4	0.54 (0.21, 0.88)^a^
Zagreb County	16	41.5	0.39 (0.20, 0.57)^b^
Varazdin County	9	23.8	0.38 (0.13, 0.62)^b^
Krapina-Zagorje County	7	18.6	0.38 (0.10, 0.65)^b^

The standard population used was the 2011 Croatian Census

*SIR* standardized incidence ratio, *CI* confidence interval

^a^ *p* < 0.05

^b^ *p* < 0.0001

^c^ The following Croatian counties correspond to the historical region of Slavonia

**Table 3:** Standardized incidence ratios for upper urothelial carcinomas in Brod-Posavina County and the City of Zagreb, 2001-2011

	Observed	Expected	SIR (95% CI)
Brod-Posavina County			
Males	36	11.9	3.03 (2.04, 4.01)^a^
Females	50	10.1	4.96 (3.59, 6.34)^a^
City of Zagreb			
Males	59	58.9	1.00 (0.74, 1.25)
Females	48	49.0	0.98 (0.70, 1.26)

The standard population used was the 2011 Croatian Census

*SIR* standardized incidence ratio, *CI* confidence interval

^a^ *p* < 0.0001

## Discussion

Our main results demonstrate a 3.9-fold increased risk for UUCs in Brod-Posavina County in comparison to Croatia during the 10-year period. Our findings were in accordance with earlier studies conducted in Croatia where BEN is prevalent [6, 11, 14]. Similarly, Cvitkovic et al. reported a 4.6-fold UUC risk in Brod-Posavina County during 2003–2009 [14]. As for the City of Zagreb, no elevated cancer risks for UUCs were noted for the total study period, even after stratifying by sex. Though the prevalence of BEN has declined over the years in Brod-Posavina County [18], the risk of UUC development remains high according to our data. This drop in prevalence may be due to either a low incidence in BEN, indicating a reduction in exposure activity, or a high case-fatality [18]. The latter can be ruled out since more and more people are living longer with BEN than in the past [18]. Today, BEN is commonly detected in the 6th decade of life [25]. Underreporting and migration could also explain some of the drops in registered BEN cases. From our map, we noted counties along the coast having greater than expected frequencies of incident UUCs. Though our findings in Dalmatia were non-significant, it is possible that residents of Slavonia sought employment in the tourism sector along the coast during the 2008 economic crisis. Unemployment in Slavonia was about 130–180% higher than the national average and an estimated 35,000 residents migrated elsewhere in the country during our study period [26].

Renal pelvic tumors are about twice as common than those of the ureter [2, 5]. Despite no differences in diagnosis, a third of all UUC cases in Croatia including Brod-Posavina County and the City of Zagreb were tumors of the ureter. Though it remains a matter of clinical debate [27, 28], several single and multicenter studies have shown ureteral tumors to have a worse prognosis than those of the renal pelvis after treatment with nephroureterectomy [29–31]. Incident cases of UUCs in Brod-Posavina County were predominately female (58%) and tended to be significantly older at the time of diagnosis than those in the City of Zagreb. Females in Brod-Posavina County were also at higher risk of developing UUCs than males when compared to their Croatian counterparts. Even after stratifying by sex, there were no elevated cancer risks in the City of Zagreb. Our findings are contrary to Western countries, where UUCs are 2 to 3 times more prevalent among males [2, 4, 5]. It appears that female predominance regarding UUC incidence is unique to endemic regions of BEN [6, 11, 14, 32]. This may be related to differences in timing of diagnosis and disease progression given that the male-to-female ratio of dying from an UUC in Brod-Posavina County is 2:1 [14].

A limitation in our study was our inability to compare incidence rates directly between counties due to an insufficient sample size. In most cases, indirect standardized rates are not comparable when age structures of populations differ [33]. SIRs can only be compared with their respective standard population and not with each other due to their given weights used to generate each ratio [33]. Additionally, county of residence was limited to time of diagnosis. Therefore, we were unable to assess the cumulative effect of length of residence in a particular county on UUC risk. This is especially of value for Brod-Posavina County, where a residence of over 20 years in an endemic household or village is required to establish a BEN diagnosis [34]. It is possible that residents of Brod-Posavina County migrated elsewhere during our study period. Given this, our SIRs for Brod-Posavina County may be underestimated. Finally, information on tumor grade and stage was unavailable to us in this study. Pathologically, both are known to influence the survival of UUC patients after treatment surgery [2, 5, 27]. In the past, lower grade and stage tumors were unique to UUC patients from BEN endemic regions resulting in a more conservative approach of treatment [13, 35]. This is no longer the case. Comparative studies of UUC patients from BEN endemic and non-endemic areas of Serbia describe a more aggressive pattern of tumor behavior in both [36, 37]. Though we no longer see differences in tumor behavior between BEN endemic and non-endemic regions [35, 38], more UUC patients from regions endemic to BEN are presenting with higher grade and stage tumors at diagnosis than ever before [35]. This may be attributed to the disease itself, changes in treatment practice, or our ability to detect it at an earlier stage. Therefore, investigations of temporal trends in tumor behavior of UUC patients in Croatia are warranted.

Conflicting BEN estimates amongst the endemic foci continue to exist [16–23]. This is, in part, due to the adaptation of different sets of diagnostic criteria over the years. Subsequently, this has led to the indiscriminate use of different parameters and cutoff values for the identification of BEN cases. Recently, a consensus statement was created in 2013 by a panel of experts from the “International Workshop on Diagnostic Criteria on Endemic Nephropathy” to address some of these issues [34]. Future epidemiologic studies should identify regional differences, question their capacity to perform BEN-UUC related research, and rule out local characteristics unique to the endemic foci as well as evaluate methods of case ascertainment prior to assuming true changes in BEN.

Our study is the first to visualize a consistently high cancer risk for UUCs during 2001–2011, especially among females in Brod-Posavina County. Although Brod-Posavina County only made up 3.7% of the total Croatian population (as of 2011), it had the highest frequency of incident UUC cases after the City of Zagreb. It is important to note, that the two counties with significant cancer risks for UUCs are in Slavonia along the Sava River and border Bosnia and Herzegovina. Furthermore, our findings reinforce the apparent geographical association between BEN and UUCs; thus, suggesting a common etiology that may be multifactorial in nature, involving both genetic and environmental factors, due to evidence of familial clustering [39] and disease development after migrating to the endemic foci, such as in the case of the Ukrainian immigrants who settled in the endemic villages of Brod-Posavina County [40]. Numerous etiological agents for BEN have been proposed over the years, including heavy metals and mycotoxins (i.e., ochratoxin A) [41]. However, none warrant enough scientific evidence for establishing causality. The most prominent risk factor for BEN is dietary exposure to aristolochic acid (AA) in bread from seeds of *Aristolochia clematitis* comingling with wheat grain [42, 43]. However, this dietary route of exposure remains questionable given the lack of temporality between the presence of ripe *A. clematitis* seeds and when wheat is harvested [44, 45] as well as the amount and time of exposure to dietary AA needed to develop BEN (i.e., eating bread daily with at least seven mature seeds of *A. clematitis* for the past seven decades) [45, 46]. Unfortunately, the role of AA in the etiology of BEN was not pursued by the scientific community until the early 1990s when a high incidence of rapidly progressive tubulointerstitial renal disease was reported among a group of Belgian women who were taking herbal dietary supplements that accidentally contained *Aristolochia fangchi*, an Aristolochia species [47]. Almost half of the women developed associated UUCs within a few years [41]. Despite the longer duration period (15–20 years) needed to develop BEN, both AA-induced nephropathy (AAN) and BEN resemble one another in terms of their clinical manifestations and pathophysiology [41, 48–50]. This has led to the hypothesis of a common etiological agent, dietary exposure to AA, which may act as an environmental risk factor for BEN and its associated UUCs. Improvements in harvesting and milling technologies have been attributed to the reduction of AA exposure and the subsequent declines in BEN incidence [18, 43, 51].

## Conclusion

In conclusion, our findings provide novel insights into the geographic distribution of UUC risk, demonstrating a 3.9-fold increased risk of UUC development, especially among females, in Brod-Posavina County when compared to Croatia as a whole, and should serve as preliminary data for future UUC and BEN-related research in Croatia. Given that many BEN patients develop associated UUCs, concurrent screening programs for UUCs and BEN should be considered, not only in endemic areas of BEN but also the surrounding rural areas and amongst at-risk groups such as those undergoing hemodialysis, who frequently develop UUCs [52, 53]. This would help to clarify BEN-UUC associations by identifying common risk factors while standardizing disease estimates across endemic regions for BEN.

## Data Availability

The datasets used and/or analysed during the current study are available from the corresponding author on reasonable request.

## Abbreviations

AA: Aristolochic acid
BEN: Balkan endemic nephropathy
CI: Confidence interval
GIS: Geographic information system
SIR: Standardized incidence ratio
UUC: Upper urothelial carcinoma
